# STIL balancing primary microcephaly and cancer

**DOI:** 10.1038/s41419-017-0101-9

**Published:** 2018-01-19

**Authors:** Dhruti Patwardhan, Shyamala Mani, Sandrine Passemard, Pierre Gressens, Vincent El Ghouzzi

**Affiliations:** 1 PROTECT, INSERM, Université Paris Diderot, Sorbonne Paris Cité, Paris, France; 2 Centre for Neuroscience, IISC Bangalore, India; 3 Curadev Pharma, B 87, Sector 83, Noida, UP 201305, India; 4 AP HP, Hôpital Robert Debré, Service de Génétique Clinique, Paris, France; 5 Centre for the Developing Brain, Division of Imaging Sciences and Biomedical Engineering, King’s College London, King’s Health Partners, St. Thomas’ Hospital, London, UK

## Abstract

Cell division and differentiation are two fundamental physiological processes that need to be tightly balanced to achieve harmonious development of an organ or a tissue without jeopardizing its homeostasis. The role played by the centriolar protein STIL is highly illustrative of this balance at different stages of life as deregulation of the human *STIL* gene expression has been associated with either insufficient brain development (primary microcephaly) or cancer, two conditions resulting from perturbations in cell cycle and chromosomal segregation. This review describes the recent advances on STIL functions in the control of centriole duplication and mitotic spindle integrity, and discusses how pathological perturbations of its finely tuned expression result in chromosomal instability in both embryonic and postnatal situations, highlighting the concept that common key factors are involved in developmental steps and tissue homeostasis.

## Facts

- STIL is a cell cycle-regulated protein specifically recruited at the mitotic centrosome to promote the duplication of centrioles in dividing cells.
- Complete loss of STIL results in no centrosomes, no cilia, and is not compatible with life.
- By contrast, residual or increased expression of STIL is viable but alters the centriole duplication process leading to either impaired or excessive centrosome formation.
- Genetic mutations in human STIL result in either residual expression or stabilization of STIL at the centrosome both leading to mitotic spindle defects and primary microcephaly (MCPH7).
- Abnormally high expression of STIL in differentiated tissues triggers centrosomal amplification and is associated with an increased metastatic potential in multiple cancers.

## Open questions

- Centrosome amplification is seen both in cancer and in MCPH phenotype. How is the context important in determining the phenotype?
- Is presence of STIL in the centrosome important in determining cell fate?
- STIL has several binding partners. To what extent is the STIL phenotype due to the independent functions of these binding partners?

## Introduction

The developing brain appears particularly sensitive to centrosome dysfunction, which is also associated with a wide range of cancers. The centrosome is a cytoplasmic organelle built around microtubule-based core components called centrioles. This review focuses on the *STIL* gene that encodes a regulatory protein necessary for centriole biogenesis and is expressed in many cell types. The structure and function of STIL is described followed by an account of two phenotypes that have been associated with STIL dysfunction, autosomal recessive primary microcephaly (MCPH), and cancer. Reasons for these different phenotypes are discussed in relation with centriole duplication and mitotic checkpoints that operate in different cellular contexts to deal with aneuploidy and chromosomal instability.

### STIL structure

**Fig. 1:**
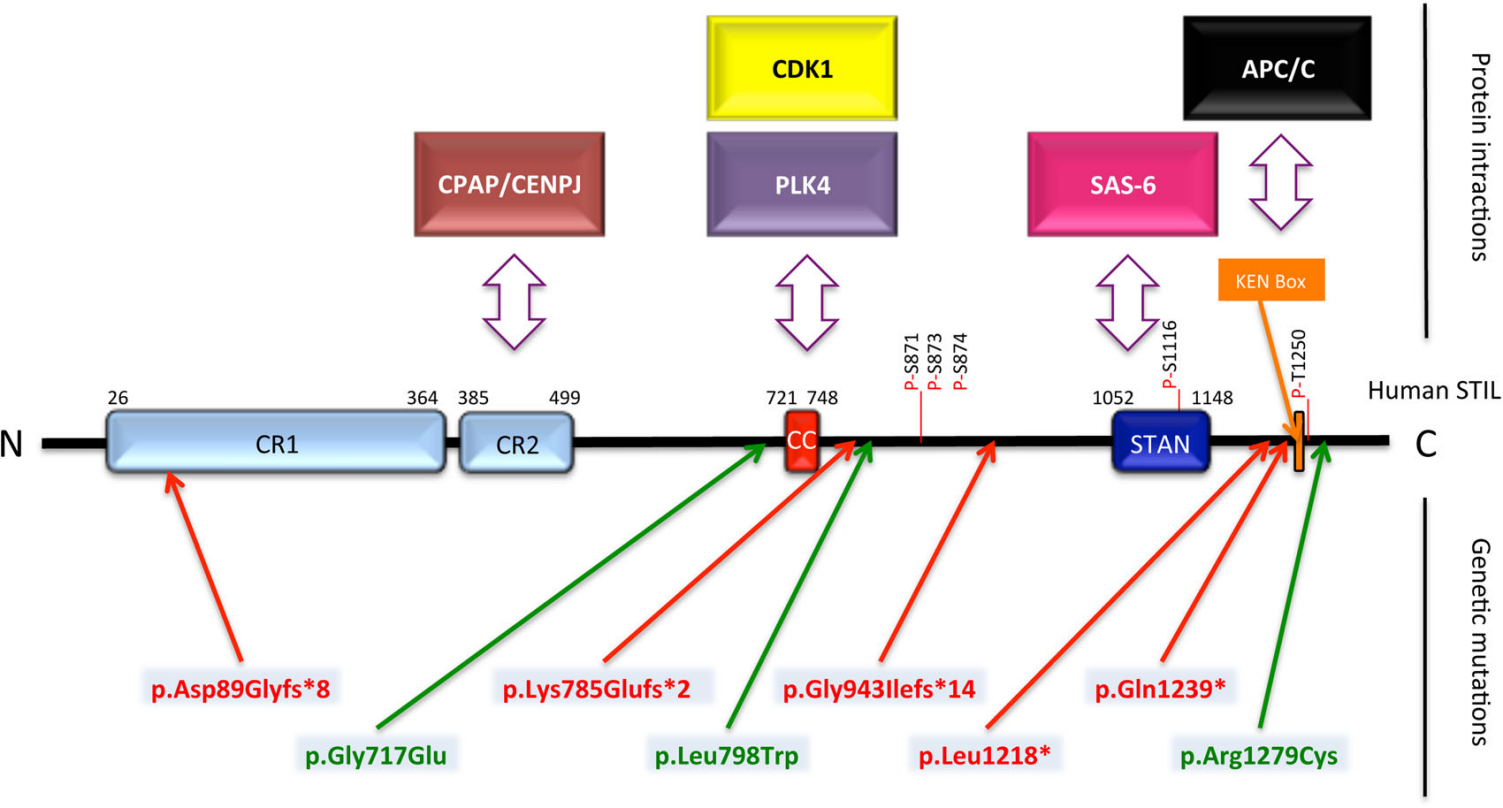
Conserved regions, functional domains, and genetic mutations in the human STIL protein Blue and orange boxes represent regions of the protein that are highly conserved across species (*CR* conserved region, *CC* Coiled-Coil domain, *STAN* STIL/Ana2 domain, *KEN Box* Conserved Lys-Glu-Asn residues). Double-headed arrows indicate known interactions with STIL. Known phosphorylated residues in 871, 873, 874, 1116, and 1250 are indicated by a red line. Human mutations predicted to truncate the protein are displayed in red and missense mutations are in green

The human *STIL* gene was initially identified in a common chromosomal rearrangement in T-cell acute lymphoblastic leukemia and named *SCL/TAL1* Interrupting Locus (*SIL/STIL*)^1^. It is predicted to encode five isoforms. Isoform1 (NP_001041631.1; henceforth referred to as *STIL*) is a 1288-amino-acid protein; isoform2 has 1287 amino acids (NP_001269865.1) and differs from isoform1 by a missing serine875; isoforms3–5 (NP_001269866.1, NP_001269867.1, NP_001269868.1) have several amino acids missing but their significance is unknown. STIL contains conserved regions and interacts with several proteins (Fig. 1). CR2 (amino acids 385–499) is a proline-rich domain that includes the conserved PRXXPXP motif, which interacts with the Centrosomal P4.1-Associated Protein (CPAP/CENPJ). The coiled-coil domain (amino acids 721–748) is important both for Polo-Like Kinase 4 (PLK4) and Cyclin Dependent Kinase 1 (CDK1 also known as CDC2)/CyclinB binding^2,3^ and for STIL oligomerization^4^. The STAN domain (amino acids 1052–1148) mediates the binding of the centriole protein SAS-6^2^. At the C terminus is the KEN box involved in Anaphase-Promoting-Complex/Cyclosome (APC/C)-mediated degradation of STIL^5^. The C terminus of STIL can also interact with conserved components of the Hedgehog signaling such as Suppressor-of-fused homolog (SUFU)^6^ and GLI1^7^.

### STIL function in centriole duplication

**Fig. 2:**
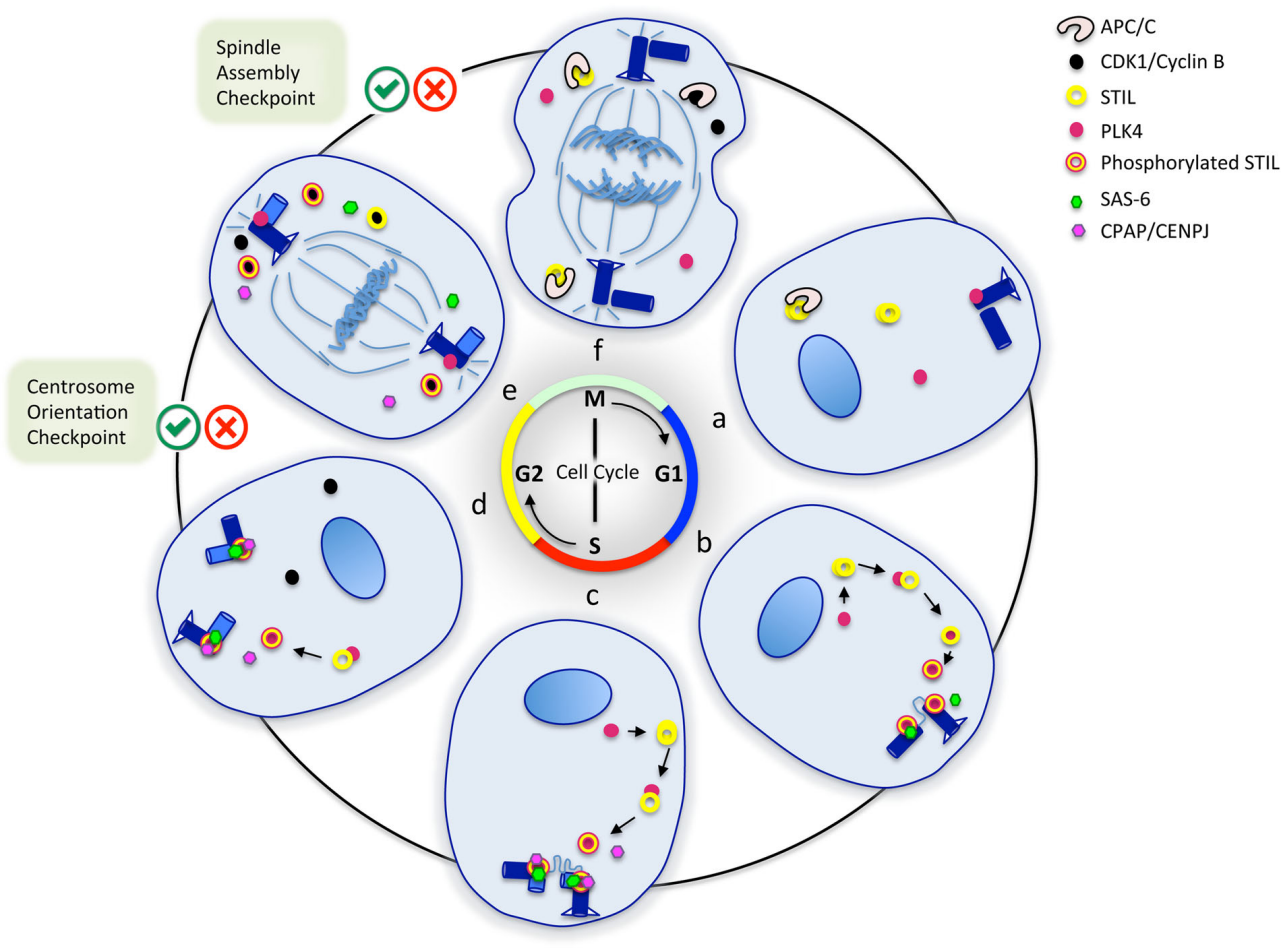
STIL regulation during the cell cycle. Six phases of the cell cycle are represented **a** Early G1 phase: STIL levels are low in the cytoplasm and STIL is absent at the centrosome. **b**, **c** G1–S and S phases: STIL levels are high in the cytoplasm and STIL starts being associated with the centrosome. PLK4 interacts and phosphorylates STIL. STIL recruits SAS-6 and CPAP to the centrosome, contributing to the assembly of the cartwheel. The procentriole starts elongating in S phase. **d** G2 phase: the two centrosomes begin to move apart. CDK1/CYCLIN B is active. Centrosome orientation checkpoint. **e** The nuclear envelope breakdown occurs, CDK1 binds to STIL and moves it to the cytoplasm, there is no more STIL at the centrosome, cartwheel disassembles. Spindle assembly checkpoint. **f** Anaphase, APC/C is fully active, cytoplasmic STIL is degraded. The cell nucleus or chromosomes with their mitotic spindle are represented in blue. Centrioles are shown in dark blue. STIL is represented by a yellow donut surrounded by a red circle when phosphorylated. PLK4 is represented by a red dot, CDK1/CYCLIN B by a black dot, SAS-6 by a small green hexagon, CPAP/CENPJ by a small pink hexagon, and APC/C by a black and white C-shape. Centrosome orientation and spindle assembly checkpoints are indicated by green/red tick boxes

The centriole is an evolutionarily conserved structure consisting of a ninefold symmetric cartwheel with microtubule triplets, and thought to have been present in the Last Eukaryotic Common Ancestor^8^. The centrosome has two centrioles orthogonal to each other, surrounded by proteins constituting the pericentriolar material (PCM). Both the centriolar core and the PCM nucleate microtubules, which are important for positioning the mitotic spindles and imparting polarity and asymmetry to the cell. During cell division the centrioles duplicate only once; STIL and its interactors are central to this process, ensuring the fidelity of this unique duplication, thereby minimizing chromosome instability^9^. STIL transiently associates with PLK4 and SAS-6 to form the core module for centriole duplication^10^. PLK4 is related to the Polo-kinase family of serine/threonine kinases and initiates centriole formation^11,12^, while SAS-6, a coiled-coil protein that self-assembles into the ninefold symmetry gives the carthweel its shape^13,14^. STIL and PLK4 are initially maintained at low levels in non-dividing and differentiated cells^15^: STIL forms dimers or tetramers through its CC domain and is degraded in the cytoplasm by APC/C and its co-activator CDC20, through recognition of the KEN motif^4,5^. STIL level in the cytoplasm rises in G1 because its degradation by APC/C is prevented by the absence of CDC20^5^. PLK4 recruits STIL at the base of the parental centriole through the CC domain and phosphorylates the STAN domain at the late G1/G1-S transition^16–18^. Phosphorylated STIL then promotes its binding to the C-terminal region of SAS-6 and recruits it to the outside wall of the centriole^2,17^. This core module along with CPAP, also recruited by STIL to the centriole, starts the assembly of the cartwheel and centriole duplication^19,20^. At early S phase, the centrosome-associated protein ROTATIN was recently shown to associate with STIL and contribute to building full-length centrioles^21^. At S–G2 transition, the centrioles are duplicated but remain close to each other, potentially preventing reduplication. Remarkably, CDK1, transiently expressed at late G2 and during M phase, competes with PLK4 for binding to the CC domain of STIL, preventing PLK4 from recruiting STIL until mitosis is completed^3,16,22^. The spindle assembly checkpoint, a highly conserved mechanism also called mitotic checkpoint, prevents the degradation of CDK1 during mitosis by the inhibition of APC/C-CDC20 until all chromosomes are properly segregated^23^. CDK1 is thought to trigger progressive dissociation of STIL and SAS-6 from early mitotic centrosomes, thereby initiating cartwheel disassembly during M phase and ensuring that centriole biogenesis occurs only once before cell divides^5^. Upon activation, APC/C-CDC20 degrades CDK1 for entry into anaphase and again marks STIL for degradation until next the G1 phase. Thus, by prometaphase there is no STIL at the centrosome and by anaphase there is no STIL in the cytoplasm either until upon mitotic exit APC/C is blocked and STIL levels builds up once again in G1 (Fig. 2).

### STIL function in SHH signal transduction

SHH is a morphogen involved in patterning, proliferation, and survial of neural stem cells during development^24^. SHH binds to its receptor Patched thereby relieving its inhibition on Smoothened and resulting in the activation of GLI transcription factors. That GLI proteins are the main downstream effectors for SHH signaling is borne out by the fact that activated GLI can rescue the loss-of-function of SHH and lead to proliferation and increased cell survival^25^. The initial findings that STIL participates in the control of SHH signaling came from Stil^−^^/^^−^ mouse embryos, which showed a marked reduction of Patched and Gli1 expression^26^. Interestingly, Stil^−^^/^^−^ mutants also lack primary cilia^27^, a structure present in almost all cell types, which is assembled beneath the plasma membrane, by the protrusion of the microtubule-based axoneme. During interphase, the mother centriole and its appendages constitute the basal body, from which the axoneme elongates^28,29^. STIL requirement for cilia formation likely stems from its function in supporting centriole biogenesis and stability. Receptors for SHH are abundant on cilia membranes allowing cilia to act as receivers of signals and as platforms where downstream effectors can be modified^30^. Thus, cilia, whose presence depends on STIL, are required for activity of the SHH pathway^31^.

**Fig. 3:**
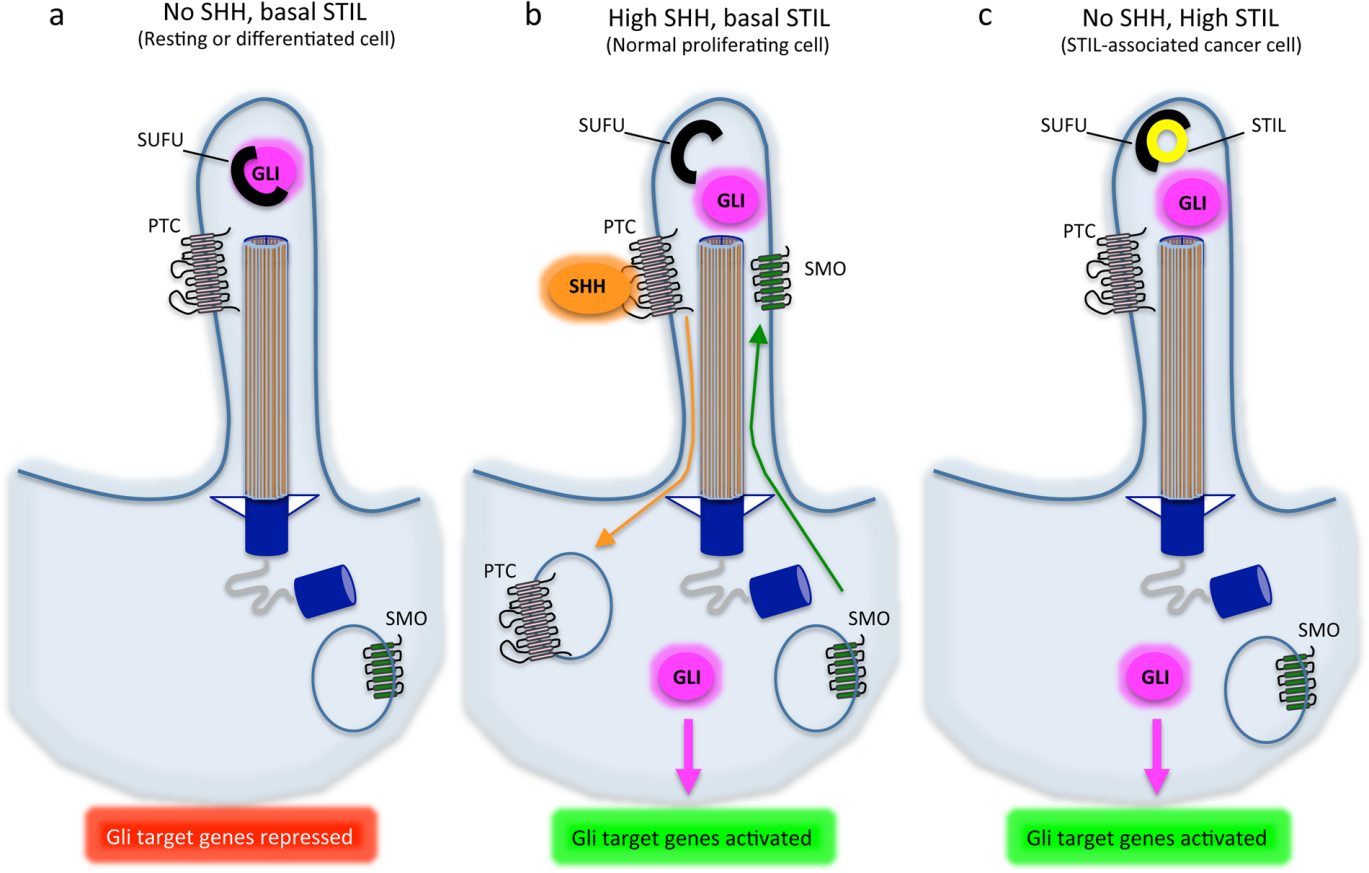
STIL and SHH signaling at the cilium A cilium and its axonemal structure are represented in three different contexts of expression of SHH and STIL. *SMO* = SMOOTHENED receptor, *PTC* = PATCHED receptor. **a** In resting or differentiated cells, there is no SHH and low STIL expressed in the cilium. The PTC receptor is expressed at the membrane while the SMO receptor is degraded in the cytoplasm. SUFU binds and tether GLI proteins in the cytoplasm, blocking GLI-dependent transcription. **b** When SHH is expressed, it binds to its receptor PTC inducing its internalization and degradation in the cytoplasm. This allows SMO expression at the membrane of the cilium and leads to GLI derepression. **c** When STIL is abnormally highly expressed during interphase, it becomes abundant in the cilium where it binds to SUFU, releasing GLI proteins and thus activating GLI-mediated transcription even in the absence of SHH

STIL also interferes with the SHH pathway in a direct manner, by interacting with SUFU. SUFU acts as a negative regulator of SHH by tethering GLI1 in the cytoplasm. STIL binds to SUFU in the cytoplasm of pancreatic cancer cells, thus releasing GLI1 for transcription of SHH-downstream genes^6^. In PC12 cells, STIL interacts with the SUFU/GLI1 complex, and its downregulation results in a decrease in both SHH signaling and cell proliferation^7^. A similar correlation has been evidenced in zebrafish retina cells, further suggesting that STIL plays a role in cell proliferation through the SHH pathway^32^ (Fig. 3).

### Consequences of STIL deregulation

Given the centrality of STIL in centrosome duplication and cilium biogenesis, deregulation of STIL is predictably profound. Stil^−^^/^^−^ embryos die at midgestation with axial midline defects, resulting from aberrant SHH signaling^26^. MEFs derived from Stil^−^^/^^−^ embryos show a marked decrease in mitotic index, and Stil knockdown results in the absence of identifiable centrosomes during interphase and multiple spindle poles with disrupted γ-tubulin signals during mitosis^33^, as well as in the loss of primary cilia^27^. Conversely, STIL overexpression causes centrosome amplification, resulting in a star-like pattern around the parental centriole, consistent with a role of STIL in the onset of procentriole formation. Accordingly, this star-like structure is positive for the centriolar proteins CP110 and centrin^20,34^. Mutations within the various domains of STIL give predictable phenotypes. When the CR2 domain or the CC domain or the STAN domain is deleted, there is no centrosomal duplication^16,18^, whereas removal of the KEN box leads to centrosome amplification due to the inability of STIL to be degraded^5^. However, both overduplication and lack of duplication of centrosomes lead to abnormal mitotic spindle assembly and consequently increase the chances of abnormal chromosomal segregation and aneuploidy.

Another consequence of STIL deregulation is the indirect effect on interacting partners. STIL binding to PLK4 supresses PLK4 auto-inhibition, thereby allowing its trans-phosphorylation and protecting activated PLK4 from degradation^35^. Conversely, depletion of STIL leads to a marked accumulation of PLK4 in an inactive conformation. Therefore, changes in STIL levels have immediate consequences on PLK4 activation levels^10,17^. STIL also negatively regulates Chfr, an E3 ligase that blocks mitotic entry in response to mitotic stress. No direct interaction between STIL and Chfr has been shown, but STIL expression results in an increase of Chfr auto-ubiquitination, thereby promoting its proteasomal degradation and proper progression of cells through mitosis^33^. Data support a role of Chfr in defective mitotic progression associated with reduced activation of CDK1/CYCLIN B and centrosomal abnormalities caused by the lack of STIL^33^. Thus, STIL deregulation likely impacts the activation level of CDK1/CYCLIN B and the timing of entry into mitosis, through its regulation of Chfr.

### STIL expression during human brain development

*STIL* is expressed in both fetal and adult tissues. However, its expression levels fluctuate with the cell cycle, making it difficult to detect in whole tissue, especially if the cells are not synchronized. Indeed, it is detected more easily in cancer cell lines and tissue where it is overexpressed^36,37^. The BrainSpan Atlas (http://www.brainspan.org/) and the BrainCloud database (http://braincloud.jhmi.edu) show its expression pattern during brain development. The association of STIL with cell proliferation is borne out by its expression pattern during fetal stages. At 15 postconceptional weeks *STIL* is strongly expressed in the ventricular and subventricular zones of the forebrain, the ganglionic eminence and the rostral migratory stream, and less expressed in intermediate zone, subplate, cortical plate, marginal zone, and subgranular layer of the forebrain. This pattern persists at 21 postconceptional weeks, although the expression is reduced in the subventricular zone. The expression of *PLK4*, *SAS-6*, and *CPAP* also broadly shows this pattern of expression, although the expression of *SAS-6* and *STIL* diverges in some regions of the cortical plate. In the cerebellum, *STIL*, *PLK4*, and *SAS-6* but not *CPAP* are expressed in the external granule layer and regions of the rhombic lip. However, none of these genes are expressed in the transient Purkinje cell cluster, the ventricular matrix zone of the cerebellum or in the migratory streams of the hindbrain. Microdissection experiments at different developmental stages confirmed that *STIL* expression is high in the ventricular zone and low in the subplate and cortical zones (http://www.brainspan.org/). Further, microdissected diencephalon did not have *STIL* (http://www.blueprintnhpatlas.org/). This suggests that (i) the structural components of the cartwheel STIL-SAS-6-PLK4 is by no means obligatory for centrosome duplication in all cells and (ii) STIL is present in specific populations of dividing cells.

### STIL mutations and primary microcephaly

**Fig. 4:**
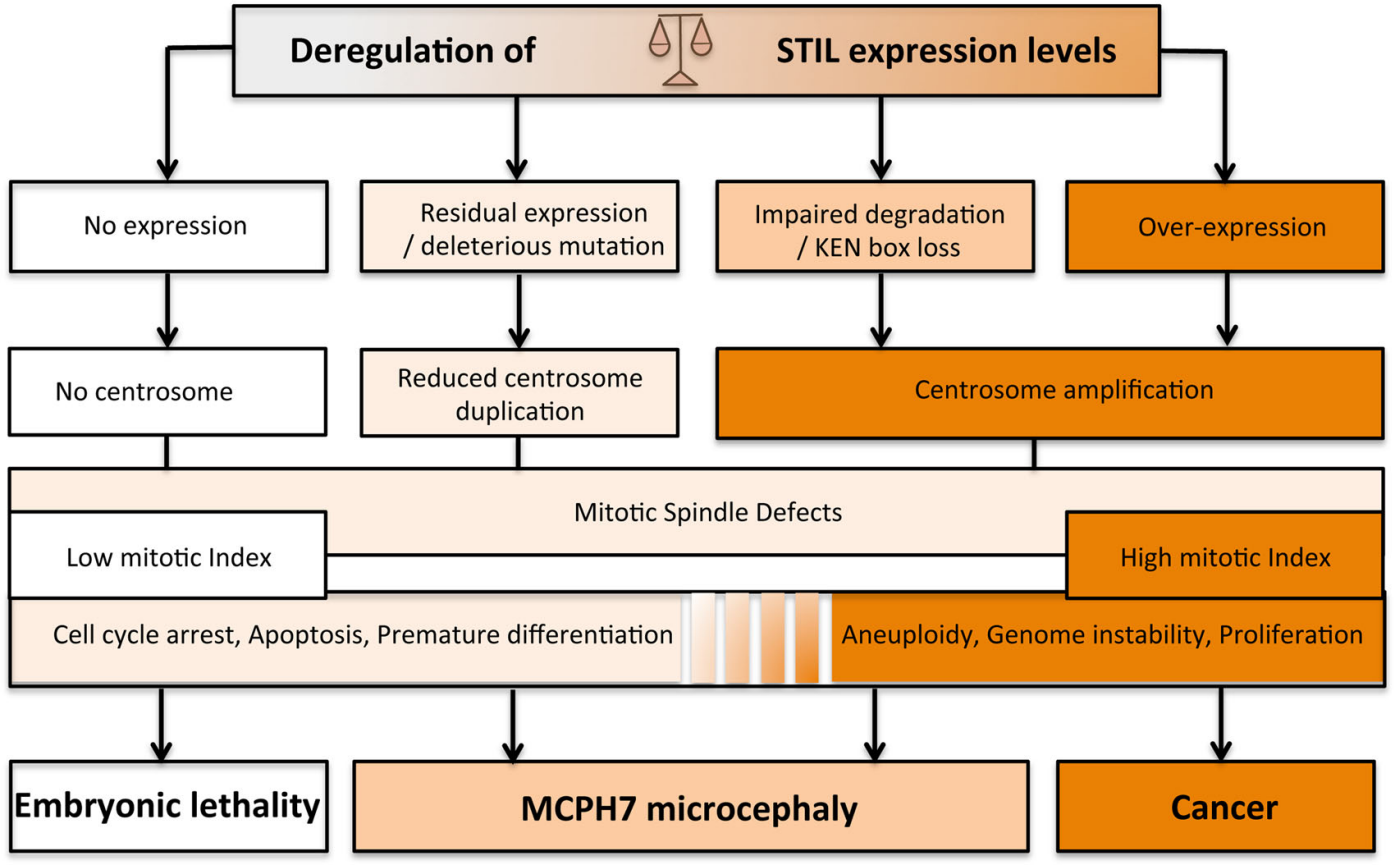
Consequences of deregulating STIL expression levels STIL expression levels are finely regulated both during development and in differentiated tissues. While total absence of STIL is lethal during development, high STIL levels due to random somatic mutations in differentiated tissues result in aneuploidy and/or high GLI-mediated transcription that can lead to cancer. Congenital deregulation of STIL (both a too high and a too weak expression) leads to microcephaly by either reduced or amplified centrosome duplication, highlighting the importance of a tightly regulated STIL expression

Microcephaly (small brain size) is indirectly diagnosed by a head circumference smaller than the age-specific and gender-adjusted mean by more than 2 standard deviations (S.D.s) at birth. Primary microcephaly refers to hereditary microcephalies already detectable *in utero*. Most of them are autosomal recessive and include (i) isolated forms called MicroCephaly Primary Hereditary (MCPH), (ii) forms associated with growth retardation, called microcephalic dwarfism. Most of *STIL* mutations identified in patients are associated with a MCPH phenotype and *STIL* is recognized as MCPH7^38–40^. However, a few patients exhibit criteria of microcephalic dwarfism, as short stature has been reported^41^. So far, eight mutations in *STIL* have been described in 37 patients all showing severe microcephaly (−4 to −10 S.D.). Five mutations are splice^42,43^, deletion, nonsense^43^, or duplication^44^ that predict the production of truncated proteins, while three mutations are missense substitutions^44–46^ (Fig. 1). Despite the different kinds of mutations, the various domains affected (Table 1), and the report of lobar holoprosencephaly and partial agenesis of the corpus callosum in addition to microcephaly in some cases^42,44,45^, no clear genotype–phenotype correlation has come out so far. However, the fact that these mutations trigger a phenotype compatible with life (although very severe) is surprising since genetic ablation of *STIL* is embryonically lethal early during embryogenesis in mice and fish^26,47^. This suggests that *STIL* mutations in human do not result in a complete loss-of-function, or are partially compensated by other genes. The first hypothesis is supported by the findings that two C-terminal nonsense mutations (p.Val1219* and p.Gln1239*), which result in the loss of the KEN box^43^, do not affect the centrosomal localization of STIL nor its functionality but rather abolish its degradation by APC/C in late mitosis^5^. Removal of the KEN box thus causes a strong accumulation of the mutant protein, resulting in centrosome amplification^5^. Alternatively, microcephaly in MCPH7 patients can result from a decrease of STIL levels. This was illustrated by rescue experiments in U2OS cells showing that the pGly717Glu mutation induces a reduced but non-null activity of STIL on centriole duplication^45^. Another mutation, inducing exon 5 skipping and frameshift in the *STIL* sequence, is likely close to a null mutation as all domains are predicted to be lost^42^. However exon skipping was only partial in the patient analyzed, suggesting some residual activity of STIL, and providing an explanation for the fact that this mutation is compatible with life and the idea that MCPH7 microcephaly can result from a decreased STIL activity. Therefore, both accumulation and impairment of STIL protein levels during cell cycle affect centriole regulation and result in microcephaly (Fig. 4).

**Table 1:** List of mutations identified in the human *STIL* gene, and their functional consequence

Mutation (nt)	Mutation (aa)	Domain affected (protein)	Functional consequence	Head OFC (S.D.)	Brain MRI data	References
c.453 + 5 G > A (splice donor)	p.Asp89Glyfs*8 (truncating)	All domains	No functional protein	−9 to −10	Short cc, simplified gyration, lobar HPE	42
c.2150 G > A	p.Gly717Glu (missense)	CC domain	Presumably inability of STIL to oligomerize and interact with PLK4—decreased centriole duplication capacity	−7 to −8	Partial cc agenesis, simplified gyration, lobar HPE	4,45
c.2354_2355dupGA	p.Lys785Glufs*2 (truncating)	Phosphorylation sites, STAN domain, KEN box	Removes critical PLK4 phosphorylation sites	26 cm at birth	Cc agenesis, simplified gyration, lobar HPE	44
c.2392 T > G	p.Leu798Trp (missense)	—	ND	NA	NA	46
c.2826 + 1 G > A (splice donor)	p.Gly943Ilefs*14 (likely truncating)	STAN domain, KEN box	Not able to duplicate centrosomes since they cannot recruit SAS6	−5	NA	27,43
c.3655delG	p.Val1219* (initially reported as p.Leu128*; truncating)	KEN box	Resistance to APC/C-mediated degradation	−4 to −10	NA	5,18,27,43
c.3715 C > T	p.Gln1239* (truncating)	KEN box	Resistance to APC/C-mediated degradation	−7 to −8	NA	18,27,43,95
c.3835 C > T	p.Arg1279Cys (missense)	C terminus	ND	26 cm at birth	Cc agenesis, simplified gyration, lobar HPE	44

### STIL deregulation and cancer

Centrosomes are essential for chromosomal stability, and abnormalities of their number, or structure affect cell division^22^. Considering the central role of STIL in maintaining centrosome integrity in highly proliferating cells, STIL has been found upregulated in several cancers of bad prognosis, including lung cancer, colon carcinoma, prostate adenocarcinoma^36^, and ovarian cancers^48^. Moreover, STIL expression is associated with an increased metastatic potential in multiple cancers^49^. As expected, STIL upregulation is associated with a high histopathological mitotic index in tumors^36^ and presumably affects the formation of mitotic spindles, as well as SHH signaling and the function of its interactors.

Although cells can proliferate and microtubule can nucleate in their absence, centrosomes are obligatory for controlling spindle orientation^50^. Loss of this control leads to spindle defects, especially in highly polarized cells, where misoriented spindles influence daughter cell positioning, possibly causing a disruption in tissue morphology^51^. Several tumor suppressor genes such as *APC*, or *VHL*, are important for stabilizing microtubules and maintaining spindle orientation^51^. Similarly, overexpression of genes such as *STIL* could act as oncogenes and lead to cancer by promoting spindle defects, although direct evidence of a link between oncogene activation and spindle misorientation is lacking. STIL overexpression, which results in supernumerary centrosomes, could lead to cancer in inducing chromosomal instability^10,52^.

The role of STIL in promoting SHH signaling could be another pathway to account for its association with cancer. Following the initial observation that SHH overexpression results in the development of basal cell carcinomas in mice^53^, a large body of literature has implicated the SHH pathway in development of various cancers including a subset of medulloblastomas^54,55^. *STIL* overexpression in pancreatic adenocarcinoma was shown to de-repress GLI1 from SUFU-mediated control and this phenotype was reversed when *STIL* was downregulated^6^. Thus, during the process of carcinogenesis, the increased expression of *STIL* promotes the transcriptional activity of GLI1, which is no longer regulated quantitatively. GLI1 upregulates genes that promote sustained proliferation, cell death resistance, stemness, angiogenesis, and genomic instability, which are hallmarks of cancer^56^. Therefore, increase in STIL expression leading to uncontrolled GLI1 de-repression likely represents a crucial step toward cancer progression.

### MCPH and cancer—two conditions associated with abnormal centrosome duplication

STIL loss-of-function results in embryonic lethality in mice, fish, and most likely in human as well. MCPH7 phenotype reflects that perturbing the tuning of *STIL* expression levels is compatible with life but triggers severe patterning phenotypes during development, including brain growth. Thus, microcephaly caused by *STIL* mutations can result from either too less centrosome duplication or too much centrosome amplification (Fig. 4). In both cases, the integrity of the mitotic spindle is compromised and cell divisions have a greater chance of chromosomal instability. Similarly, the association of *STIL* upregulation with various cancers reflects a deregulation of STIL expression levels in specific tissues contributing to genomic instability. What results in MCPH rather than cancer could be the difference in mitotic checkpoints in different contexts^13,57^. Centrosome orientation checkpoint ensures that the centrosomes are duplicated and appropriately positioned before entry into mitosis and several centrosomal proteins such as CNN and SAS-6 are part of this checkpoint^58^. This checkpoint may not be present in all cells at all developmental stages but rather in those cases where the cell division plane has important outcomes^59^. In germline stem cells, for example, the division plane determines the distribution of cell fate determinants in daughter cells, helding cells at the G2–M phase until their mitotic spindles are properly oriented^58^. *STIL* downregulation results in disorganized mitotic spindles resulting in a loss of spindle orientation control^47^. In cortical development, *STIL* mutations leading to spindle orientation defects likely result in a depletion of cortical progenitors due to cell death or premature differentiation and give rise to a MCPH phenotype^57^ (Fig. 4). *PLK4* overexpression also results in centrosome amplification and aneuploidy leading to a reduction in brain size due to cell death^60^. Apoptosis inhibition in this background causes the accumulation of aneuploid cells unable to proliferate efficiently, leading to premature neuronal differentiation^60^, whereas in a P53^−^^/^^−^ context, *PLK4* overexpression results in aneuploidy and skin cancer^61^.

The spindle assembly checkpoint is the next checkpoint once cells have entered mitosis and formed spindles. In the absence of bipolar spindles, cells are held-up in prometaphase and do not proceed to anaphase^58^, which results in increased apoptosis^62^. However, cells can increase their time in mitosis, thus adapting to this checkpoint and can assemble bipolar spindles in the absence of centrosomes or in the presence of supernumerary centrosomes. First in the absence of centrosome duplication cells manage to have bipolar spindles even though there is no centriole attached to one of the poles^34^. Second is a phenomena of centrosome clustering, wherein multiple centrosomes are clustered to enable the formation of bipolar spindles, a phenomenon whose efficiency could be tissue-specific^60^. These divisions often result in aneuploidy. When centrosome integrity is compromised, cells usually arrest in G1 and do not re-enter cell cycle after a prolonged cytokinesis to avoid chromosomal instability^22^ and in many cases proliferation in the absence of centrioles additionally requires the suppression of P53^17^. Upregulation of *STIL* giving rise to aneuploidy could play a major role in providing an evolving genome to help the cells adapt to the changing environment of the cancer and escape normal checkpoints. In the developing cortex, abnormal mitotic spindles due to *STIL* mutations would result in cell fate switches rather than cell hyperproliferation. However, that patients whose *STIL* mutation is clearly associated with centrosome amplification could be at risk of developing cancer cannot be ruled out.

Seventeen MCPH loci have been described so far^40^, several of which also play a role in cancer. MCPH1 is an early DNA damage response (DDR) protein and *MCPH1* deletions have been identified as a risk for breast cancer^63^. Centrosome amplification is commonly seen following DNA damage and can arise as part of the DDR^64^. The serine-threonine kinase CHK1 plays a major role in DDR by halting cell division in G2/M until repair proteins are recruited at lesion sites. In this context, MCPH1 deficiency potentiates CHK1 activity and increases centrosome amplification^65^. Thus, compromising mitotic checkpoints results in cell division in the presence of abnormal centrosomes^63,65^. How CHK1 overactivation leads to centrosome amplification is not fully understood, but it is thought to activate CDK2, a cyclin-dependent kinase required for centrosome duplication, likely through activating its phosphorylation^66,67^. WD repeat-containing protein62 (WDR62), whose deficiency is associated with MCPH2^68–70^, is required for maintaining spindle and centrosome integrity. Its overexpression, coincident with centrosome amplification, is also seen in lung adenocarcinomas and ovarian cancers^71,72^. WDR62 interacts physically during the cell cycle with the abnormal spindle-like microcephaly-associated protein (ASPM), whose deficiency causes the most frequent primary microcephaly (MCPH5)^73^. Here too, increased ASPM levels cause tumor growth and are seen in medulloblastomas while its reduction causes a decrease in tumor proliferation^74^. CDK5RAP2 (MCPH3) is also involved in the DDR; it functions by arresting cells before mitotic entry due to imparied centrosomes but also interacts with BUB1 and MAD2, which are important in spindle activation checkpoint^75,76^. Interestingly, a correlation between centrosomal abnormalities, aneuploidy, and cytogenic risk profile is seen in acute myeloid leukemia, where gene expression profiling has revealed the differential expression of genes encoding centrosomal and mitotic spindle proteins^77^. Among these are pericentrin, a scaffold protein that anchors many other proteins at the centrosome^78^ and NuMA, which associates with dynein and microtubules to create localized pulling forces, thus regulating the correct assembly and positioning of the mitotic spindle^79^. The role of such factors points to mitotic centrosomal abnormalities as important component of cancer progression.

STIL could also have an indirect effect on cancer, as a downstream effector of PLK4. Increased PLK4 expression has been reported in malignancies such as colorectal cancer^80^, pediatric medulloblastoma^81^, and breast tumors^82^. Its transient overexpression leads to centrosome overduplication and in a P53^−^^/^^−^ background that inhibits apoptosis, it results in aneuploidy and spontaneous skin cancer^61^. Another recent study has convincingly shown that PLK4 overexpression leads to spontaneous tumors in several organs^83^. It remains to be seen whether this phenotype is dependent on STIL expression. STIL binds to PLK4 in the cytoplasm and therefore STIL expression levels could impact PLK4 cytoplasmic activity^17^, which functions to remodel the cytoskeleton and may be important for cancer invasion and metastasis as its depletion is correlated with an increase in E-cadherin expression and less metastasis^84^. CYCLIN B is also frequently found elevated in primary breast cancer, esophageal squamous cell carcinoma, laryngeal squamous cell carcinoma, and colorectal carcinoma^85–89^ and its expression indicates a bad prognosis and is correlated with the malignancy of gynecological cancers^90^. Downregulation of *STIL* decreases CDK1/CYCLIN B activity, prevents G2–M transition, and causes inhibition of tumor growth in vivo^91^. Conversely, increasing STIL could promote CDK1/CYCLIN B activity and indirectly participate in CYCLIN B-dependent proliferation in tumor cells. Absence of STIL also results in an upregulation of Chfr, and lowers PLK1, resulting in the activation of the CDC25c phosphatase. This pathway could thus control the entry of cells into mitosis independent of its obligatory role in centriole duplication^33^.

## Conclusion

*STIL* mutations in MCPH show that centrosomes and cilia are essential for normal brain development. During evolution, one way that the cortex has undergone expansion is likely linked to mechanisms controlling spindle orientation and keeping the balance between asymmetric and symmetric cell divisions^92,93^. A change in the nature of spindle orientation control has been proposed to account for the population of subventricular zone progenitors in human development and based on the strong expression of *STIL* in the ventricular zone, one may speculate that STIL is important for controlling spindle orientation in giving rise to subventricular zone’s cells^94^. Deficiency of several centrosomal proteins results in MCPH, and centrosomal proteins may thus be a key to discovering the mechanism of expansion of cortical area^38^. In addition, the involvement of STIL in cancer shows that the result of spindle defects can be vastly different depending on the stage of development and the tissue involved that probably is partly due to differential responses to mitotic checkpoint mechanisms. STIL intersects several pathways, and lowering its level could block the effects of *PLK4* overexpression, CDK1/CYCLIN B activation, and GLI1 signaling, and reduce proliferation even in the presence of aneuploidy. Thus, centrosomal proteins may be a new class of targets for cancer.
